# Factors Associated with Use of HIV Prevention and Health Care Among Transgender Women — Seven Urban Areas, 2019–2020

**DOI:** 10.15585/mmwr.mm7120a1

**Published:** 2022-05-20

**Authors:** Kathryn Lee, Lindsay Trujillo, Evelyn Olansky, Taylor Robbins, Christine Agnew Brune, Elana Morris, Teresa Finlayson, Dafna Kanny, Cyprian Wejnert, National HIV Behavioral Surveillance among Transgender Women Study Group, National HIV Behavioral Surveillance among Transgender Women Study Group, Narquis Barak, Kathleen A. Brady, Sarah Braunstein, Jasmine Davis, Sara Glick, Andrea Harrington, Jasmine Lopez, Yingbo Ma, Aleks Martin, Genetha Mustaafaa, Tanner Nassau, Gia Olaes, Jennifer Reuer, Alexis Rivera, William T. Robinson, Ekow Kwa Sey, Sofia Sicro, Brittany Taylor, Dillon Trujillo, Erin Wilson, Pascale Wortley

**Affiliations:** ^1^Division of HIV Prevention, National Center for HIV, Viral Hepatitis, STD, and TB Prevention, CDC; ^2^Social & Scientific Systems, Inc., Atlanta, Georgia.; CrescentCare; Philadelphia Department of Public Health; New York City Department of Health and Mental Hygiene; CrescentCare; University of Washington, School of Medicine, Division of Allergy and Infectious Diseases, Public Health - Seattle & King County, HIV/STD Program; Philadelphia Department of Public Health; New York City Department of Health and Mental Hygiene; Los Angeles County Department of Public Health; Public Health - Seattle & King County, HIV/STD Program; Georgia Department of Public Health; Philadelphia Department of Public Health; Los Angeles County Department of Public Health; Washington State Department of Health; New York City Department of Health and Mental Hygiene; Louisiana State University Health Science Center in New Orleans – School of Public Health, Louisiana Office of Public Health STD/HIV/Hepatitis Program; Los Angeles County Department of Public Health; San Francisco Department of Public Health; Georgia Department of Public Health; San Francisco Department of Public Health; San Francisco Department of Public Health; Georgia Department of Public Health

Transgender women* are disproportionately affected by HIV. Among 1,608 transgender women who participated in CDC’s National HIV Behavioral Surveillance (NHBS) during 2019–2020, 42% received a positive HIV test result (*1*). This report provides results from seven U.S. urban areas where the 2019–2020 NHBS questionnaire was administered. Thirty-eight percent of participants reported having previously received a positive test result for HIV. Detrimental socioeconomic factors, including low income (44%), homelessness (39%), and severe food insecurity in the past 12 months (40%), were common and associated with lower receipt of HIV prevention and treatment services. Having a usual health care source or a provider with whom the participant was comfortable discussing gender-related health issues was associated with improved HIV prevention and treatment outcomes, including HIV testing, preexposure prophylaxis (PrEP) use, and viral suppression. These findings illustrate the benefit of gender-affirming approaches used by health care providers (*2*), and highlight the challenging socioeconomic conditions faced by many transgender women. Ensuring access to gender-affirming health care approaches and addressing the socioeconomic challenges of many transgender women could improve access to and use of HIV prevention and care in this population and will help achieve the goals of the Ending the HIV Epidemic in the United States initiative (*3*).

Initiated in 2003, NHBS conducts biobehavioral surveillance among persons at high risk for HIV infection. During June 2019–February 2020, NHBS surveyed 1,608 transgender women in seven U.S. urban areas using respondent-driven sampling.^†^ Eligible participants^§^ completed an interviewer-administered questionnaire and were offered an HIV test. The questionnaire included measures of gender identity,^¶^ income, health insurance, housing,** food insecurity,^††^ HIV status, viral suppression (if HIV-positive), comfort with their health care provider in discussing gender-related health issues (hereafter referred to as comfort with a provider), unmet need for health care,^§§^ and usual source of health care. Because of racial and ethnic disparities in HIV prevalence, recruitment was focused on Black or African American and Hispanic or Latina transgender women as initial sampling recruits. Incentives were provided for completion of the interview and HIV test. Adjusted prevalence ratios (aPRs) and 95% CIs for prevention and treatment outcomes, by self-reported HIV status, were estimated using log-linked Poisson regression models with generalized estimating equations clustered on recruitment chain and urban area; models were adjusted for age, race and ethnicity, and urban area. Analyses were conducted using SAS software (version 9.4; SAS Institute). This activity was reviewed by CDC and was conducted consistent with applicable federal law and CDC policy.^¶¶^

Data from 1,608 transgender women were included in this analysis (Table 1). Thirty-eight percent reported having previously received a positive HIV test result.*** Forty-four percent earned <$10,000 annually. During the past 12 months 39% experienced homelessness, and 40% experienced severe food insecurity. Nearly one third (31%) of participants were interviewed in Los Angeles. By urban area, reports of homelessness ranged from 22% to 59%, and reports of recent severe food insecurity ranged from 28% to 47%. Comfort with a provider varied by urban area from 66% to 91%.

**TABLE 1:** Structural and health care factors among transgender women (N = 1,608)* — National HIV Behavioral Surveillance System, seven U.S. urban areas, 2019–2020

Characteristic	No. (%)							
	Transgender women	Severe food insecurity ^§^	Nights homeless^†^				Has usual source of care^¶^	Comfort with a health care provider when discussing gender-related issues
			365	30–364	<30	None		
**Age group, yrs**								
18–29	496 (30.9)	244 (49.2)	49 (9.9)	135 (27.2)	57 (11.7)	247 (49.8)	374 (75.4)	357 (72.0)
30–39	461 (28.7)	186 (40.4)	48 (10.4)	105 (22.8)	44 (9.5)	258 (56.0)	372 (80.7)	344 (74.6)
40–49	307 (19.1)	113 (36.8)	23 (7.5)	57 (18.8)	23 (7.5)	192 (62.5)	270 (88.0)	254 (82.7)
≥50	343 (21.3)	94 (27.4)	32 (9.3)	41 (12.0)	15 (4.4)	238 (69.4)	308 (89.8)	295 (86.0)
Race and ethnicity**								
Black, non-Hispanic	569 (35.4)	221 (38.8)	63 (11.1)	124 (21.8)	51 (9.0)	321 (56.4)	469 (82.4)	452 (79.4)
Hispanic or Latina^††^	643 (40.0)	275 (42.8)	49 (7.6)	122 (19.0)	61 (9.5)	396 (61.6)	532 (82.7)	481 (74.8)
White, non-Hispanic	180 (11.2)	81 (45.0)	25 (13.9)	39 (21.7)	13 (7.2)	98 (54.4)	150 (83.3)	148 (82.2)
Multiple, non-Hispanic	124 (7.7)	44 (35.5)	8 (6.5)	39 (31.5)	9 (7.3)	60 (48.4)	105 (84.7)	107 (86.3)
Other,^§§^ non-Hispanic	89 (5.5)	15 (16.9)	6 (6.7)	13 (14.6)	6 (6.7)	60 (67.4)	66 (74.2)	61 (68.5)
**Gender identity^¶¶^**								
Woman	509 (31.7)	199 (39.1)	57 (11.2)	118 (23.1)	37 (7.3)	287 (56.4)	431 (84.7)	407 (80.0)
Man	6 (0.4)	—***	—	—	—	—	5 (83.3)	—
Transgender woman	1,404 (87.3)	558 (39.7)	131 (9.3)	295 (21.0)	126 (9.0)	817 (58.2)	1,144 (81.5)	1,084 (77.2)
Transgender man	11 (0.7)	—	—	—	—	7 (63.6)	9 (81.8)	6 (54.6)
A gender not listed here	94 (5.9)	40 (42.6)	12 (12.8)	24 (25.5)	7 (7.5)	46 (48.9)	74 (78.7)	64 (68.1)
**Currently has health insurance**								
Yes	1,337 (83.2)	512 (38.3)	120 (9.0)	281 (21.0)	104 (7.8)	794 (59.4)	1,178 (88.1)	1,127 (84.3)
No	270 (16.8)	124 (45.9)	32 (11.9)	56 (20.7)	36 (13.3)	142 (52.6)	146 (54.1)	124 (45.9)
**Unmet need for health care during the past 12 months**								
Yes	323 (20.1)	186 (57.6)	37 (11.5)	97 (30.0)	36 (11.2)	147 (45.5)	238 (73.7)	224 (69.4)
No	1,285 (79.9)	451 (35.1)	115 (9.0)	241 (18.8)	104 (8.1)	789 (61.4)	1,087 (84.6)	1,027 (79.9)
**Self-reported HIV status^†††^**								
HIV–positive	615 (38.3)	229 (37.2)	60 (9.8)	139 (22.6)	50 (8.1)	350 (56.9)	546 (88.8)	537 (87.3)
HIV–negative or unknown	991 (61.6)	407 (41.1)	92 (9.3)	199 (20.1)	89 (9.0)	585 (59.0)	778 (78.5)	714 (72.1)
**Education**								
Less than high school	347 (21.6)	168 (48.4)	35 (10.1)	75 (21.6)	33 (9.5)	192 (55.3)	283 (81.6)	268 (77.2)
High school diploma or equivalent	596 (37.1)	247 (41.4)	64 (10.7)	136 (22.8)	61 (10.2)	326 (54.7)	480 (80.5)	447 (75.0)
Some college or technical degree	486 (30.2)	181 (37.2)	40 (8.2)	105 (21.6)	33 (6.8)	290 (59.7)	416 (85.6)	395 (81.3)
College degree or more	177 (11.0)	39 (22.0)	13 (7.3)	21 (11.9)	12 (6.8)	128 (72.3)	144 (81.4)	140 (79.1)
**Annual household income, USD**								
40,000–74,999	173 (10.8)	25 (14.5)	—	9 (5.2)	13 (7.5)	145 (83.8)	145 (81.8)	140 (80.9)
20,000–39,999	274 (17.0)	78 (28.5)	22 (8.0)	42 (15.3)	20 (7.3)	186 (67.9)	228 (83.2)	218 (79.6)
10,000–19,999	435 (27.1)	155 (35.6)	29 (6.7)	83 (19.1)	30 (6.9)	274 (63.0)	372 (85.5)	358 (82.3)
≤9,999	711 (44.2)	373 (52.5)	94 (13.2)	201 (28.3)	76 (10.7)	324 (45.6)	571 (80.3)	523 (73.6)
**Urban area**								
Atlanta, Georgia	132 (8.2)	55 (41.7)	12 (9.1)	37 (28.0)	18 (13.6)	62 (47.0)	88 (66.7)	87 (65.9)
Los Angeles, California	504 (31.3)	224 (44.4)	50 (9.9)	136 (27.0)	43 (8.5)	270 (53.6)	420 (83.3)	374 (74.2)
New Orleans, Louisiana	165 (10.3)	77 (46.7)	12 (7.0)	35 (21.2)	11 (6.7)	106 (64.2)	143 (86.7)	136 (82.4)
New York, New York	279 (17.4)	114 (40.9)	21 (7.5)	46 (16.5)	27 (9.7)	181 (64.9)	245 (87.8)	222 (79.6)
Philadelphia, Pennsylvania	220 (13.7)	61 (27.7)	13 (5.9)	35 (15.9)	19 (8.6)	151 (68.6)	174 (79.1)	200 (90.9)
San Francisco, California	198 (12.3)	77 (38.9)	39 (19.7)	37 (18.7)	15 (7.6)	80 (40.4)	179 (90.4)	160 (80.8)
Seattle, Washington	110 (6.8)	29 (26.4)	5 (4.6)	12 (10.9)	7 (6.4)	86 (78.2)	76 (69.1)	72 (65.5)
**Total**	**1,608 (100)**	**637 (39.6)**	**152 (9.5)**	**338 (21.0)**	**140 (8.7)**	**936 (58.2)**	**1,325 (82.4)**	**1,251 (77.8)**

**Abbreviation: **USD = U.S. dollars.

* Numbers might not sum to totals because of missing data.

^†^ Homelessness was defined as having lived on the street, in a shelter, in a single room occupancy hotel, or in a car during the past 12 months.

^§^ Severe food insecurity was defined as not eating for a whole day because there wasn't enough money for food at some point during the past 12 months.

^¶^ Usual source of care was defined as having a place to go when sick or in need of health advice other than a hospital emergency department.

** Because of racial and ethnic disparities in HIV prevalence, recruitment was focused on Black or African American and Hispanic or Latina transgender women.

^††^ Hispanic or Latina transgender women might be of any race.

^§§^ Includes persons who indicated Asian, American Indian or Alaska Native, or Native Hawaiian or other Pacific Islander race.

^¶¶^ Participants were asked to report their current gender identities from the following response options: woman, man, transgender woman, transgender man, or a gender not listed here. All eligible participants reported a gender identity of “woman” or “transgender woman;” however, participants were able to select more than one response option. Gender identities are not mutually exclusive.

*** Dashes indicate suppression because of small cell size (<5).

^†††^ Participants who reported having a previous positive HIV test result were defined as self-reported HIV–positive.

Socioeconomic status and health care accessibility were associated with health outcomes (Table 2). Among participants who reported a previous positive test result for HIV, self-reported viral suppression was less common among participants who reported experiencing homelessness during the past 12 months (aPR = 0.88; p = 0.003), and the likelihood of viral suppression decreased as the number of nights of homelessness increased. Severe food insecurity (aPR = 0.84; p<0.001) and unmet need for health care (aPR = 0.89; p = 0.027) were also less common among participants who reported viral suppression. Comfort with a provider (aPR = 1.17; p = 0.007) was more common among participants who reported viral suppression. Similar associations were found for current use of antiretroviral medication. Having a usual source of health care was also associated with current use of antiretroviral medication (aPR = 1.16; p = 0.015).

**TABLE 2:** HIV treatment among transgender women living with a positive HIV test result — National HIV Behavioral Surveillance System, seven U.S. urban areas,* 2019–2020

Characteristic	No. of transgender women	Viral suppression			Current antiretroviral use		
		No. (%)	aPR^†^ (95% CI)	p-value	No. (%)	aPR^†^ (95% CI)	p-value
**Annual household income, USD**							
40,000–74,999	51	45 (88.2)	1.12 (1.00–1.25)	0.043	48 (94.1)	1.06 (0.99–1.15)	0.107
20,000–39,999	94	83 (88.3)	1.18 (1.09–1.27)	<0.001	88 (93.6)	1.07 (1.01–1.14)	0.023
10,000–19,999	177	129 (72.9)	0.96 (0.87–1.05)	0.365	165 (93.2)	1.08 (1.02–1.14)	0.012
≤9,999	290	209 (72.1)	Ref	—	249 (85.9)	Ref	—
**Education**							
Less than high school	144	108 (75.0)	Ref	—	130 (90.3)	Ref	—
High school diploma or equivalent	236	171 (72.5)	1.02 (0.92–1.12)	0.735	210 (89.0)	1.00 (0.95–1.05)	0.967
Some college or technical degree	196	155 (79.1)	1.08 (0.98–1.19)	0.127	177 (90.3)	1.02 (0.95–1.08)	0.606
College degree or more	39	33 (84.6)	1.18 (1.03–1.34)	0.013	34 (87.2)	0.98 (0.88–1.08)	0.661
**Experienced homelessness^§^**							
Yes	265	179 (67.6)	0.88 (0.81–0.96)	0.003	226 (85.3)	0.91 (0.88–0.96)	<0.001
No	350	288 (82.3)	Ref	—	325 (92.9)	Ref	—
**No. of nights homeless^§^**							
365	60	33 (55.0)	0.75 (0.58–0.96)	0.025	47 (78.3)	0.84 (0.76–0.93)	0.001
30–364	139	97 (69.8)	0.91 (0.83–1.00)	0.048	119 (85.6)	0.92 (0.87–0.98)	0.011
<30	50	39 (78.0)	1.02 (0.88–1.18)	0.804	47 (94.0)	0.99 (0.91–1.08)	0.799
None	350	288 (82.3)	Ref	—	325 (92.9)	Ref	—
**Severe food insecurity^¶^**							
Yes	229	150 (65.5)	0.84 (0.76–0.92)	<0.001	193 (84.3)	0.92 (0.87–0.96)	0.001
No	386	317 (82.1)	Ref	—	328 (92.7)	Ref	—
**Currently has health insurance**							
Yes	560	435 (77.7)	1.14 (0.96–1.35)	0.133	507 (90.5)	1.16 (1.03–1.30)	0.016
No	54	32 (59.3)	Ref	—	43 (79.6)	Ref	—
**Unmet need for health care during the past 12 months**							
Yes	90	58 (64.4)	0.89 (0.81–0.99)	0.027	74 (82.2)	0.90 (0.84–0.97)	0.008
No	525	409 (77.9)	Ref	—	477 (90.9)	Ref	—
Has usual source of care**							
Yes	546	420 (76.9)	1.07 (0.94–1.22)	0.323	496 (90.8)	1.16 (1.03–1.32)	0.015
No	69	47 (68.1)	Ref	—	55 (79.7)	Ref	—
**Comfort with a health care provider^††^**							
Yes	537	423 (78.8)	1.17 (1.04–1.32)	0.007	490 (91.2)	1.16 (1.05–1.29)	0.004
No	78	44 (56.4)	Ref	—	61 (78.2)	Ref	—
**Total**	**615**	**467 (75.9)**	—	—	**551 (89.6)**	—	—

**Abbreviations:** aPR = adjusted prevalence ratio; Ref = referent group; USD = U.S. dollars.

* The seven urban areas include Atlanta, Georgia; Los Angeles, California; New Orleans, Louisiana; New York, New York; Philadelphia, Pennsylvania; San Francisco, California; and Seattle, Washington.

^† ^Adjusted for age, race and ethnicity, city, and network size and clustered on urban areas and recruitment chains.

^§^ Homelessness was defined as having lived on the street, in a shelter, in a single room occupancy hotel, or in a car during the past 12 months.

^¶^ Severe food insecurity was defined as not eating for a whole day because there was not enough money for food at some point during the past 12 months.

** Usual source of care was defined as having a place to go when sick or in need of health advice other than a hospital emergency department.

^††^ Comfort with a health care provider was defined as having a health care provider with whom the participant is comfortable discussing gender-related health issues.

Among participants who did not report a previous positive test result for HIV, testing for HIV during the past 12 months was more likely among those who reported having a usual source of health care (aPR = 1.16; p<0.001) and comfort with a provider (aPR = 1.12; p = 0.004) (Table 3). PrEP use was more common among participants who reported having health insurance (aPR = 1.54; p<0.001), a usual source of health care (aPR = 2.54; p<0.001), and comfort with a provider (aPR = 1.79; p<0.001), and less likely among participants who reported an unmet need for health care (aPR = 0.82; p = 0.050). PrEP use was also more common among participants who had experienced severe food insecurity than those who had not (aPR = 1.23; p = 0.024).

**TABLE 3:** HIV prevention services among transgender women without known HIV infection — National HIV Behavioral Surveillance System, seven U.S. urban areas,* 2019–2020

Characteristic	No. of transgender women	HIV test in the past 12 months			PrEP use in the past 12 months		
		No. (%)	aPR^†^ (95% CI)	p-value	No. (%)	aPR^†^ (95% CI)	p-value
**Annual household income, USD**							
40,000–74,999	122	93 (76.2)	0.93 (0.85–1.01)	0.099	23 (18.8)	0.73 (0.53–0.99)	0.043
20,000–39,999	180	136 (75.6)	0.90 (0.82–0.98)	0.022	55 (30.6)	1.09 (0.90–1.32)	0.377
10,000–19,999	258	214 (82.9)	0.99 (0.94–1.04)	0.640	96 (37.2)	1.45 (1.22–1.74)	<0.001
≤9,999	421	358 (85.0)	Ref	—	113 (26.8)	Ref	—
**Education**							
Less than high school	203	173 (85.2)	Ref	—	51 (25.1)	Ref	—
High school diploma or equivalent	360	283 (78.6)	0.93 (0.86–1.01)	0.067	110 (30.6)	1.26 (1.02–1.56)	0.033
Some college or technical degree	290	244 (84.1)	1.00 (0.94–1.07)	0.944	91 (31.4)	1.27 (0.97–1.66)	0.087
College degree or more	138	106 (76.8)	0.95 (0.85–1.06)	0.379	36 (26.1)	1.06 (0.81–1.40)	0.662
**Experienced homelessness^§^**							
Yes	406	349 (86.0)	1.10 (0.99–1.21)	0.076	126 (31.0)	1.08 (0.93–1.25)	0.332
No	586	458 (78.2)	Ref	—	162 (27.6)	Ref	—
**No. of nights homeless^§^**							
365	92	73 (79.3)	1.03 (0.90–1.17)	0.663	24 (26.1)	0.98 (0.70–1.38)	0.899
30–364	199	176 (88.4)	1.12 (1.00–1.25)	0.059	62 (31.2)	1.05 (0.84–1.32)	0.654
<30	90	78 (86.7)	1.10 (0.99–1.21)	0.073	29 (32.2)	1.09 (0.83–1.43)	0.525
None	586	458 (78.2)	Ref	—	162 (27.6)	Ref	—
**Severe food insecurity^¶^**							
Yes	408	342 (83.8)	1.02 (0.96–1.10)	0.495	137 (33.6)	1.23 (1.03–1.47)	0.024
No	582	463 (79.5)	Ref	—	149 (25.6)	Ref	—
**Currently has health insurance**							
Yes	777	638 (82.1)	1.06 (0.98–1.16)	0.155	240 (30.9)	1.54 (1.26–1.88)	<0.001
No	216	170 (78.7)	Ref	—	48 (22.2)	Ref	—
**Unmet need for health care during the past 12 months**							
Yes	233	190 (81.6)	0.99 (0.93–1.05)	0.792	60 (25.7)	0.82 (0.68–1.00)	0.050
No	760	618 (81.3)	Ref	—	228 (30.0)	Ref	—
Has usual source of care**							
Yes	779	650 (83.4)	1.16 (1.08–1.23)	<0.001	261 (33.5)	2.54 (1.86–3.45)	<0.001
No	210	154 (73.3)	Ref	—	26 (12.4)		—
**Comfort with a health care provider^††^**							
Yes	714	601 (84.2)	1.12 (1.04–1.21)	0.004	240 (33.6)	1.79 (1.43–2.24)	<0.001
No	274	206 (75.2)	Ref	—	48 (17.5)	Ref	—
**Total**	**991**	**786 (82.3)**	—	—	**288 (29.0)**	—	—

**Abbreviations:** aPR = adjusted prevalence ratio; PrEP = preexposure prophylaxis; Ref = referent group; USD = U.S. dollars.

* The seven urban areas include Atlanta, Georgia; Los Angeles, California; New Orleans, Louisiana; New York, New York; Philadelphia, Pennsylvania; San Francisco, California; and Seattle, Washington.

^† ^Adjusted for age, race and ethnicity, city, and network size and clustered on urban areas and recruitment chains.

^§ ^Homelessness was defined as having lived on the street, in a shelter, in a single room occupancy hotel, or in a car during the past 12 months.

^¶^ Severe food insecurity was defined as not eating for a whole day because there was not enough money for food at some point during the past 12 months.

** Usual source of care was defined as having a place to go when sick or in need of health advice other than a hospital emergency department.

^††^ Comfort with a health care provider was defined as having a health care provider with whom the participant is comfortable discussing gender-related health issues.

## Discussion

Experiencing homelessness, poverty, and food insecurity was common among transgender women and might result from the pervasive experience of stigma and discrimination, which reduce access to education, employment, and health care (*4*). These structural factors are associated with lower likelihood of viral suppression among transgender women with HIV infection. When a person experiences challenges securing food or housing, prioritization of HIV treatment might be interrupted (*5*). Facilitating transgender women’s access to interventions that address socioeconomic conditions, such as the U.S. Department of Housing and Urban Development’s Housing Opportunities for Persons with AIDS (HOPWA) program,^†††^ could help ensure that basic needs are met and improve the health of persons with HIV in this population.

Despite existence of need-based programs like the Ryan White HIV/AIDS Program^§§§^ and Ready, Set, PrEP,^¶¶¶^ results indicate that participants without health insurance or with an unmet need for health care were less likely to achieve viral suppression or report PrEP use. Evaluation of these and similar programs might help identify barriers to participation that need to be addressed to ensure that persons in need are aware of and accessing these programs.

Having a usual source of health care and comfort with a provider were associated with a higher likelihood of viral suppression, HIV testing, and PrEP use, all of which play key roles in HIV prevention. Comfort with a provider can help alleviate the stigma and discrimination that often deter transgender persons from seeking care (*6*). Perceived interactions with hormones, concerns about side effects, medical mistrust, competing priorities, and the belief that PrEP is specifically for gay men are all documented barriers to PrEP use among transgender persons (*7*). A gender-affirming provider can help transgender women overcome barriers to PrEP use.

The findings in this report are subject to at least four limitations. First, the results are not representative of all transgender women residing outside the seven urban areas. Second, the data are self-reported and are subject to recall and social desirability biases. Third, the findings reported here are associations, and causality cannot be inferred. Finally, gender-affirming health care is a complex, multifaceted construct (*8*), and is not fully described by the measure of comfort with a provider when discussing gender-related health issues that was used in this analysis.

Early detection of HIV, appropriate treatment, and proven prevention interventions are effective tools in the fight against HIV and are key strategies for ending the HIV epidemic (*3*). The findings in this report highlight an additional need for health care providers and other public health officials to ensure appropriate levels of cultural competency when providing services for transgender persons. Providers can use CDC’s Patient-Centered Care for Transgender People: Recommended Practices for Health Care Settings**** as a starting point for understanding how to provide affirming services. Although access to health insurance and gender-affirming health care is critical to connecting transgender women to HIV prevention and care services; access to food, housing, and income are also essential.

**Summary**

**What is already known about this topic?**

Transgender women are disproportionately affected by HIV.

**What is added by this report?**

During 2019–2020, 38% of transgender women surveyed in seven major U.S. cities reported receiving a previous positive HIV test result. Low income (44%), experiencing homelessness (39%), and severe food insecurity (40%) were common and associated with lower likelihood of receipt of HIV prevention and health care; having a health care provider with whom the participant is comfortable was positively associated with receiving those services.

**What are the implications for public health practice?**

Ensuring access to basic needs, such as housing, food, and income, and providing gender-affirming health care could improve access to and use of HIV prevention and treatment services by transgender women.

## Footnotes

* Persons who were assigned male sex at birth and who currently identify as women or transgender women.

† Respondent-driven sampling is a methodology similar to snowball sampling and is often used when trying to sample hard-to-reach populations. The method relies on multiple waves of peer-to-peer recruitment to achieve the desired sample size. https://www.jstor.org/stable/10.1525/sp.2002.49.1.11?seq=1

§ Eligible persons were those who were aged ≥18 years, had current residence in a participating urban area, had not previously participated in the current survey cycle, had ability to complete the survey in either English or Spanish, provided informed consent, and reported a gender identity of woman or transgender woman and were assigned male sex or intersex at birth.

¶ Participants were asked to report their current gender identity from the following response options: woman, man, transgender woman, transgender man, or a gender not listed here. Participants were able to select more than one response option.

** Participants were asked if they had experienced homelessness during the past 12 months, including living on the street, in a shelter, in a single room occupancy hotel, or in a car. They were also asked to provide the number of nights during the past 12 months that they experienced homelessness.

†† Severe food insecurity was defined as having not eaten for a whole day because there was not enough money for food at some point during the past 12 months.

§§ Having an unmet need for care was defined as a “time when you needed medical care but didn’t get it because you couldn’t afford it” during the past 12 months.

¶¶ 45 C.F.R. part 46.102(l)(2), 21 C.F.R. part 56; 42 U.S.C. Sect. 241(d); 5 U.S.C. Sect. 552a; 44 U.S.C. Sect. 3501 et seq.

*** Among participants, 38% self-reported living with HIV during the interview and were asked questions related to HIV treatment. During postinterview HIV testing, an additional 4% of participants received a positive HIV test result, for a total of 42% of participants who received a positive HIV test result (https://www.cdc.gov/hiv/library/reports/hiv-surveillance.html). Those who did not report living with HIV during the interview were not asked about HIV treatment.

†††
https://www.hud.gov/hudprograms/hopwa

§§§
https://ryanwhite.hrsa.gov/

¶¶¶ https://www.hiv.gov/federal-response/ending-the-hiv-epidemic/prep-program

****
https://www.cdc.gov/hiv/clinicians/transforming-health/health-care-providers/affirmative-care.html
